# *Populus ussuriensis PuWRKY22* Transcription Factor Activates the ABA Receptor PYL4 to Enhance Drought Resistance

**DOI:** 10.3390/plants14172621

**Published:** 2025-08-23

**Authors:** Qiuhui Wang, Danni Li, Lihua Yang, Yu Yang, Shuchao Huang, Yipeng Zhao, Qingjie Guan

**Affiliations:** Key Laboratory of Restoration and Reconstruction of Saline-Alkali Vegetation, Ministry of Education, School of Life Sciences, Northeast Forestry University, Harbin 150040, China; wangqiuhui@nefu.edu.cn (Q.W.); 18204622990@163.com (D.L.); 18315847959@nefu.edu.cn (L.Y.); 2024122963@nefu.edu.cn (Y.Y.); huangshuchao@nefu.edu.cn (S.H.); zypsy@nefu.edu.cn (Y.Z.)

**Keywords:** *Populus ussuriensis* K., WRKY, drought stress, ABA signaling pathway

## Abstract

Drought stress poses a significant threat to tree growth, making the development of drought-resistant species essential for ecological restoration. WRKY transcription factors are critical regulators of plant drought responses; however, the role of *WRKY22* in the woody species *Populus ussuriensis* K. remains unclear. In this study, the *PuWRKY22* gene was cloned from *P. ussuriensis* via homologous cloning and was found to be highly expressed in leaves and responsive to abscisic acid (ABA) signaling. Subcellular localization confirmed that PuWRKY22 is a nuclear protein. Using fluorescein enzyme complementation assays, PuWRKY22 was shown to bind specifically to W-box cis-elements, indicating its function as a transcriptional regulator. Under ABA and osmotic (sorbitol) stress, the seed germination rate, root growth, and biomass of tobacco and *Populus davidiana × Populus bolleana* strains overexpressing *PuWRKY22* were significantly increased. Additionally, these overexpressed strains exhibited a reduction in reactive oxygen species (ROS) accumulation and a decrease in membrane lipid peroxidation. Transcriptomic analyses revealed that *PuWRKY22* activates expression of the ABA receptor gene *Ptr.PYL4* (Potri.006G104100.v4.1), which regulates stomatal closure to minimize water loss. Consistent with this, stomatal observations and photosynthetic measurements demonstrated that *PuWRKY22* enhances drought tolerance by protecting photosystem II and preserving chlorophyll content. Collectively, this study elucidates the molecular mechanism by which *PuWRKY22* enhances drought resistance in woody plants through ABA signaling, providing a foundation for breeding drought-tolerant forest species.

## 1. Introduction

As the largest terrestrial carbon sink, global forests—accounting for approximately 45% of land-based carbon reserves [1]—are increasingly threatened by climate-induced aridification, which jeopardizes their ecological functions. Data from the Global Drought

Information System (GDIS, https://www.drought.gov/international (accessed on 18 September 2024)) indicate that shifting precipitation patterns driven by El Niño events could double agricultural water demand and reduce freshwater availability by 50% by 2050 [2]. Since the 1980s, prolonged drought has placed over 280 million hectares of forest in high-risk zones for mortality, as reflected by a sharp increase in the Drought Mortality Risk (DMR) index [3,4]. This alarming trend underscores the urgent need to decipher the molecular mechanisms underlying plant drought resistance and to develop genetically resilient forest tree germplasm capable of withstanding intensifying environmental stress.

Woody plants have evolved sophisticated drought-tolerance mechanisms over long-term natural selection, with abscisic acid (ABA) serving as a central regulator of stress responses [5]. Recent studies have established that ABA signal transduction is primarily mediated through a core signaling cascade involving the PYR/PYL/RCAR receptor family. Upon drought perception, ABA binds to these receptors, inducing conformational changes that inhibit PP2C phosphatases. This inhibition releases SnRK2 kinases from repression, enabling their activation [6]. Activated SnRK2s phosphorylate downstream transcription factors—particularly members of the AREB/ABF family—thereby initiating the expression of drought-responsive genes and triggering a cascade of physiological adaptations [7]. One of the most immediate physiological outcomes of ABA signaling is the regulation of stomatal aperture. SnRK2-mediated phosphorylation activates key ion channels on guard cell membranes, including the anion channel SLAC1 [8] and the outward potassium channel GORK [9], leading to membrane depolarization, efflux of K^+^ and anions, loss of turgor pressure, and ultimately, stomatal closure. This minimizes transpirational water loss under drought conditions. Simultaneously, ABA promotes the production of reactive oxygen species (ROS) via the activation of the NADPH oxidase RBOHF, which acts as a secondary messenger to further enhance stomatal closure [10]. Beyond stomatal regulation, ABA enhances oxidative stress tolerance through a dual mechanism. It directly induces the expression of antioxidant enzymes, such as superoxide dismutase (SOD), catalase (CAT), and ascorbate peroxidase (APX) [11], and stimulates the ascorbate–glutathione cycle, enabling efficient scavenging of ROS accumulated under drought stress [12]. In addition, ABA signaling plays a critical role in maintaining the photochemical efficiency of photosystem II (PSII) under drought conditions. On one hand, ABA-induced stomatal closure limits CO_2_ availability [13], increasing the risk of PSII overexcitation and photoinhibition. On the other hand, ABA enhances non-photochemical quenching (NPQ) capacity by upregulating early light-induced proteins (ELIPs) and PsbS, thereby dissipating excess light energy and protecting PSII [14]. Furthermore, ABA facilitates the recycling of chlorophyll during leaf senescence by regulating NYC1/NOL-mediated chlorophyll degradation [15], which not only reduces photooxidative damage but also reallocates carbon and nitrogen resources to support stress adaptation. Notably, under prolonged drought conditions, ABA coordinates a PSII repair cycle [16] and NYC-dependent chlorophyll turnover [17], forming a synergistic regulatory network that dynamically balances photoprotection and resource redistribution. Together, these multifaceted processes establish a robust, multi-layered drought defense system that enables woody plants to endure and adapt to long-term water scarcity.

The WRKY transcription factor family—one of the largest and most functionally diverse in plants—plays a central role in orchestrating ABA-mediated stress responses. WRKY proteins are defined by a highly conserved WRKYGQK motif [18] and a zinc-finger domain that enable specific binding to W-box cis-elements (TTGAC(C/T)) within the promoters of target genes [19]. Through this interaction, WRKYs regulate a wide array of genes involved in environmental stress responses, forming an integral component of the ABA-responsive transcriptional network. Emerging evidence highlights the involvement of WRKY factors in modulating ABA signaling at multiple levels. For instance, *WRKY54* has been shown to directly interact with the ABA receptor *PYL8*, thereby enhancing plant sensitivity to ABA and amplifying downstream responses [20]. Such findings underscore the function of WRKY transcription factors as dynamic molecular switches that bridge environmental stress perception with ABA signal transduction, contributing to the fine-tuned regulation of drought resistance in plants.

In response to the urgent need for ecological restoration in China’s arid northern regions, this study focused on *P. ussuriensis*, an important native tree species, and identified for the first time the central regulatory role of the *PtrWRKY22* homolog *PuWRKY22* in the ABA signaling pathway. Through systematic analysis of the molecular mechanisms by which *PuWRKY22* regulates both the antioxidant defense system and ABA signal transduction, this research elucidates the genetic basis underlying drought-tolerant phenotypes in woody plants. The findings provide valuable genetic resources for the molecular breeding of drought-resistant tree species and offer significant practical implications for promoting vegetation recovery and constructing ecological barriers in drought-prone regions.

## 2. Results

### 2.1. Homologous Cloning and Functional Analysis of the PuWRKY22 Gene in P. ussuriensis

To identify the ABA-responsive WRKY transcription factors involved in drought stress regulation, transcriptomic data from *Populus trichocarpa* (v4.1) were analyzed following hormone treatments for 3 h. Heatmap analysis of differentially expressed genes revealed that *PtrWRKY22* (Gene ID: *Potri.002G164400*) exhibited strong transcriptional upregulation in response to abscisic acid (ABA) treatment (Supplementary Figure S1). Given the central role of ABA in mediating drought signaling and the established function of WRKY transcription factors in stress response pathways, *PtrWRKY22* was selected as a candidate gene for further investigation. Using a homology-based cloning strategy, the *PuWRKY22* gene—which is homologous to *PtrWRKY22*—was successfully isolated from *P. ussuriensis*. This cloning established the foundation for the subsequent functional validation of *PuWRKY22* in ABA signaling and drought resistance pathways in woody plants.

The tissue-specific expression and stress-responsive patterns of the *PuWRKY22* gene in *P. ussuriensis* were investigated using quantitative real-time PCR (qRT-PCR). As shown in Supplementary Figure S2, *PuWRKY22* exhibited the highest basal expression in leaves, suggesting its potential role in regulating foliar stress responses. Under abiotic stress treatments, *PuWRKY22* expression displayed distinct temporal and spatial dynamics. Upon exposure to 5 μM ABA, expression in stems was significantly upregulated, indicating responsiveness to ABA-mediated signaling. Under 200 mM sorbitol (osmotic stress), *PuWRKY22* expression showed a transient induction, peaking at 12 h post-treatment before declining, suggesting early activation in response to dehydration stress. These results demonstrate that *PuWRKY22* is both tissue-specific and stress-inducible, with dynamic expression profiles depending on the nature and duration of the external stimulus, supporting its involvement in adaptive stress signaling in woody plants.

**Figure 1:**
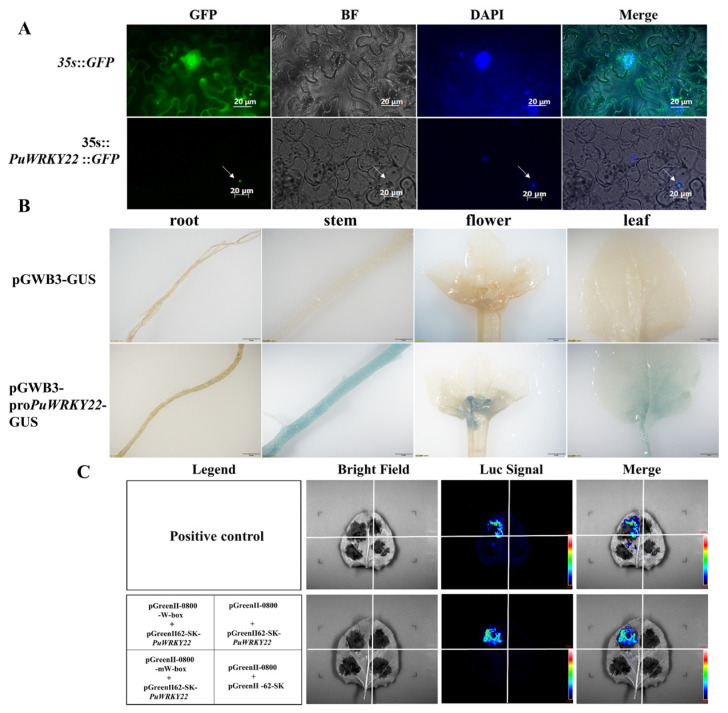
Functional analysis of *PuWRKY22*. (**A**) Subcellular localization of the PuWRKY22 protein in tobacco leaf cells. Green fluorescence from 35S::GFP served as the control, while 35S::*PuWRKY22*-GFP represents the experimental group. Nuclei were stained with DAPI. Scale bar = 20 μm. (**B**) Histochemical GUS staining of various tissues (roots, stems, leaves, flowers) in transgenic tobacco plants expressing the pGWB3-pro*PuWRKY22*::GUS construct. Scale bar = 2 mm. (**C**) Luciferase complementation assay demonstrating the binding of PuWRKY22 to the W-box element in tobacco leaves. Schematic diagram: *Agrobacterium*-infiltrated tobacco leaf regions. Bright field: light microscopy images. Luc signal: luminescence images indicating luciferase activity. Merge: overlay of bright field and luciferase signals.

Using fluorescence microscopy, the transient expression of the PuWRKY22-GFP fusion protein, driven by the 35S promoter, was observed in tobacco leaf cells. The green fluorescence signal of PuWRKY22-GFP protein was predominantly localized to the nucleus and overlapped precisely with the DAPI nuclear stain (Figure 1A). These observations are consistent with the subcellular localization prediction generated by Plant-mPLoc, confirming that the PuWRKY22 protein is a nuclear-localized transcription factor likely functioning within the cell nucleus.

Analysis of the *PuWRKY22* promoter sequence using online cis-acting element prediction tools revealed the presence of multiple regulatory elements associated with abiotic stress and hormone responses. These include the abscisic acid-responsive element (ABRE), the low-temperature response element (LTR), and the salicylic acid-responsive element (TCA), among others. These findings suggest that *PuWRKY22* may participate in diverse abiotic stress and hormone signaling pathways. Furthermore, the Pro*PuWRKY22* promoter expression vector was observed to show a blue color in tissue sites such as the roots, stems, leaves, and flowers of tobacco through GUS staining (Figure 1B), indicating that the *PuWRKY22* promoter effectively drives gene expression across multiple tissues. This experimental evidence confirms the promoter’s transcriptional activity and supports the bioinformatic predictions.

To determine whether the PuWRKY22 protein specifically binds the W-box cis-acting element, a recombinant plasmid, pGreenII 62-SK-*PuWRKY22*, was constructed and transformed into *Agrobacterium tumefaciens*. Fluorescence complementation assays were performed via *Agrobacterium*-mediated transient expression in tobacco leaves. As a positive control, fluorescence signals were detected at the injection site, confirming the validity of the reagents and experimental procedures. Co-infection of pGreenII 0800-W-box-Luc (containing three tandem repeats of the W-box element) with pGreenII 62-SK-*PuWRKY22* resulted in a strong luciferase luminescence signal (Figure 1C), demonstrating that *PuWRKY22* binds specifically to the W-box sequence and may regulate downstream gene expression. In contrast, no luminescence signal was observed when pGreenII 0800-mW-box-Luc (containing mutated W-box elements) was co-infected with pGreenII 62-SK-*PuWRKY22*, indicating that PuWRKY22 does not interact with the mutated motif. Collectively, these results confirm that PuWRKY22 functions as a typical WRKY transcription factor with specific binding affinity for the canonical W-box motif “TTGAC(C/T)”.

### 2.2. Analysis of Drought Tolerance of Overexpressed Tobacco Lines

**Figure 2:**
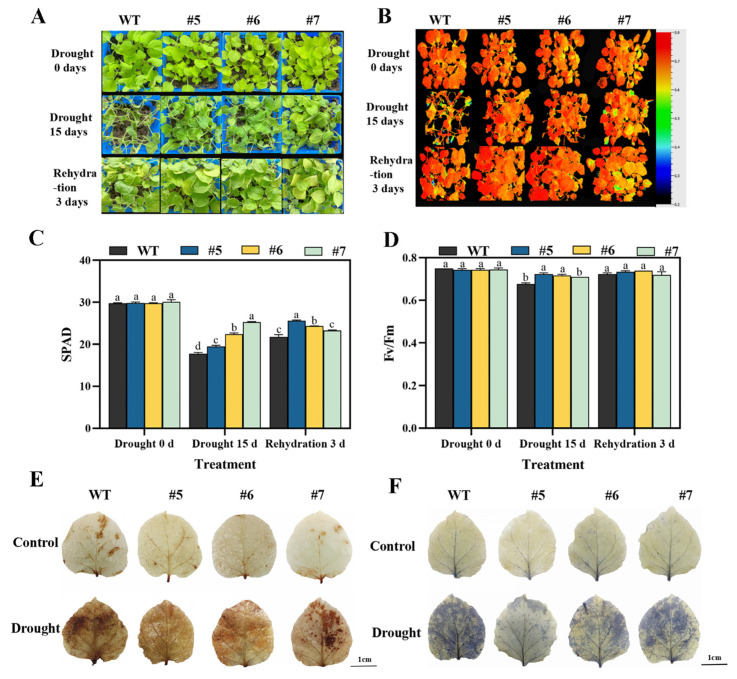
Analysis of photosynthetic characteristics and ROS levels of overexpressed tobacco under natural drought stress. (**A**,**B**) Phenotype and Fv/Fm images of tobacco at seedling stage with overexpression of *PuWRKY22* under natural drought stress, cursor range: 0.2~0.8. (**C**) Determination of chlorophyll content of tobacco. (**D**) Determination of Fv/Fm value of tobacco. (**E**,**F**) Histochemical staining of tobacco leaves. DAB staining phenotype of tobacco leaves on the left, NBT staining phenotype of tobacco leaves on the right, bar: 1 cm. Note: Data are presented as mean ± SD. Bars labeled with different letters differ significantly (one-way ANOVA, Tukey’s HSD test, *p* < 0.05).

Transgenic tobacco lines (#5, #6, and #7) exhibiting high expression levels of *PuWRKY22* were selected for drought tolerance assays. As shown in Supplementary Figures S3–S6, during the germination and three-leaf periods of tobacco, under different concentrations and stresses, overexpressed tobacco outperformed wild-type (WT) tobacco in terms of germination rate, root length, and freshness, and the effects were more significant. By observing the conditions of overexpressed and WT tobacco on the 0th and 15th days of natural drought treatment during the seedling stage and the 3rd day of rewatering treatment, it was found that overexpressed plants showed better growth status, photosynthetic efficiency (Fv/Fm value) (Figure 2A,B), and chlorophyll content (Figure 2C,D) under the coercion of sorbitol and ABA, indicating that *PuWRKY22* can reduce the damage of photosystem II and maintain photosynthesis. Furthermore, reactive oxygen species (ROS) measurements revealed significantly reduced accumulation of hydrogen peroxide (H_2_O_2_) and superoxide anions (O_2_^−^) in transgenic lines relative to the WT (Figure 2E,F). This suggests that *PuWRKY22* contributes to enhanced oxidative stress tolerance by promoting more effective ROS scavenging. Collectively, these findings demonstrate that *PuWRKY22* significantly enhances drought resilience in tobacco by modulating both photosynthetic stability and antioxidative defense pathways.

### 2.3. Analysis of Drought Tolerance of Overexpressed (OE) P. davidiana × P. bolleana Strains

**Figure 3:**
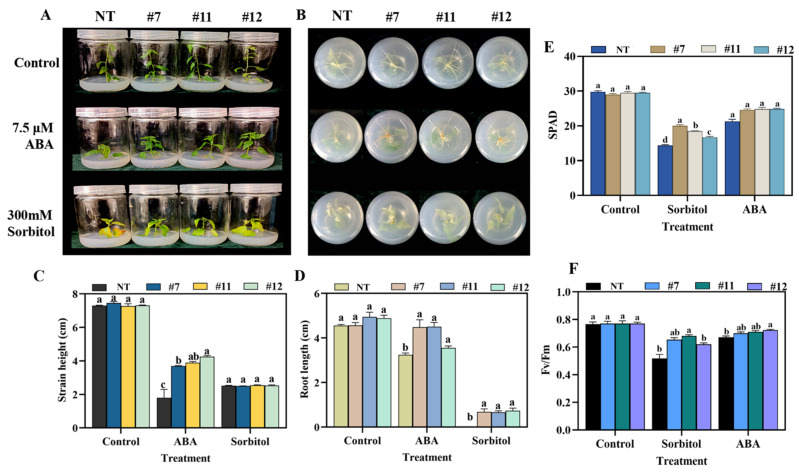
Phenotypic analysis of overexpressed *P. davidiana × P. bolleana* under ABA- and sorbitol-induced drought stress. (**A**) Representative phenotypes of natural-type and overexpressed lines after 25 days of treatment with 7.5 μM ABA and 300 mM sorbitol. (**B**) Root growth comparison under drought-simulating conditions. (**C**) Quantification of plant height. (**D**) Quantification of root length. (**E**) Chlorophyll content measured by SPAD values. (**F**) Photosystem II maximum photochemical efficiency (Fv/Fm) measurements. Note: Data are presented as mean ± SD. Bars labeled with different letters differ significantly (one-way ANOVA, Tukey’s HSD test, *p* < 0.05).

**Figure 4:**
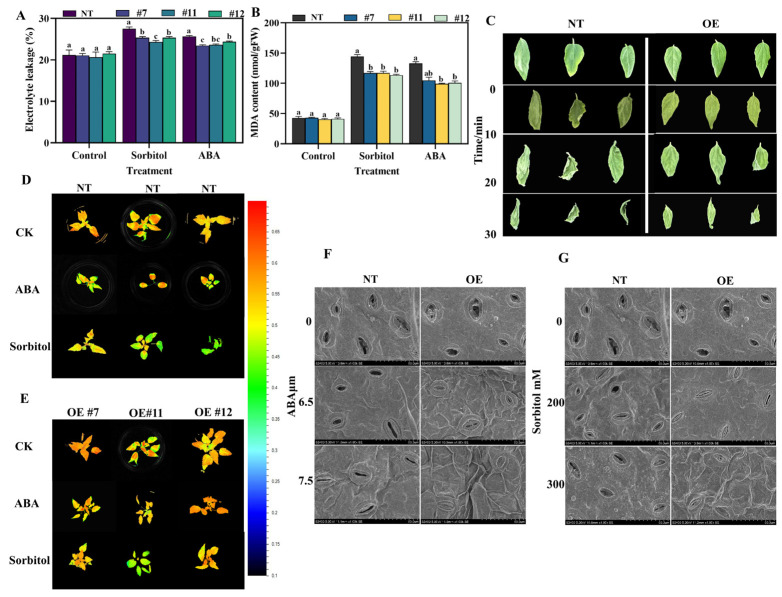
Physiological measurements and dehydration response under ABA- and sorbitol-induced drought stress in *overexpressed P. davidiana × P. bolleana* lines. (**A**) Electrolyte permeability measurement. (**B**) Malondialdehyde (MDA) content measurement. (**C**) Leaf dehydration assay showing curling phenotypes at different time points under room temperature. (**D**) Chlorophyll fluorescence (Fv/Fm) imaging of natural-type control plants under various stress treatments (the cursor range is between 0.1 and 0.7). (**E**) Chlorophyll fluorescence (Fv/Fm) imaging of overexpressed strains under various stress treatments. (**F**) Scanning electron microscopy (SEM) images of stomatal structures under ABA stress. (**G**) SEM images of stomatal structures under sorbitol stress (scale bars = 50 μm). Note: Data are presented as mean ± SD. Bars labeled with different letters differ significantly (one-way ANOVA, Tukey’s HSD test, *p* < 0.05).

The natural-type (NT) and overexpressed *P. davidiana × P. bolleana* lines exhibiting comparable growth were cultured on WPM medium supplemented with 7.5 μM ABA or 300 mM sorbitol. The WPM medium without added stress treatment was used as the control. After 25 days, growth phenotypes, plant height, and root length were measured. Under ABA-induced drought simulation, overexpressed (OE) lines displayed significantly enhanced growth compared to natural (NT) lines, as evidenced by greater plant height and longer roots (Figure 3A). In contrast, 300 mM sorbitol treatment severely inhibited growth in both types; however, natural-type plants showed almost no root development. The root length of the overexpressed strain was restricted, but it was still significantly longer than that of the natural-type (Figure 3B) one. Control plants grew normally. Further physiological assessments revealed that overexpressed plants exhibited higher plant height, root length, chlorophyll content, and Fv/Fm values than natural-type controls (Figure 3C–F). Based on the above findings, this study also conducted measurements of electrolyte leakage and malondialdehyde (MDA) content, and found that the overexpressed plants were significantly superior to the NT plants (Figure 4A,B). These results suggest that the overexpression of *PuWRKY22* enhances drought tolerance in *P. davidiana* × *P. bolleana* by improving growth capacity, photosynthetic efficiency, and reducing membrane lipid peroxidation under drought-mimicking conditions.

### 2.4. Response to Dehydration and Stomatal Changes in Overexpressed and Natural-Type Strains

To assess the dehydration response of OE and NT plants, three leaves of uniform size from tissue-cultured seedlings were detached and left at room temperature for 0, 10, 20, and 30 min, with photographs taken at each time point. As shown in Figure 4C, NT leaves exhibited pronounced curling—a hallmark of water stress—whereas OE leaves displayed significantly reduced curling, indicating enhanced tolerance to dehydration. To validate these findings, tissue-cultured *P. davidiana × P. bolleana* seedlings were divided into two groups: natural-type controls subjected to no stress; lines exposed to 7.5 μM ABA, or 300 mM sorbitol; and overexpressed lines under the same treatments. Each had three biological replicates. After 20 days of growth under 25 °C light conditions, phenotypic and chlorophyll fluorescence imaging were performed. Fluorescence images under the Fv/Fm mode (Figure 4D,E) showed significantly lower signal intensity in natural-type leaves compared to overexpressed leaves after stress treatments, indicating more severe photoinhibition and damage to photosystem II reaction centers in natural-type plants. These stressed plants were subsequently harvested for transcriptomic analysis and stomatal detection. Stomatal behavior was further examined via scanning electron microscopy following treatment with ABA and sorbitol. Under non-stress conditions, both natural-type and overexpressed plants had similarly open stomata with comparable apertures and uniform stomatal distribution. However, under increasing ABA and sorbitol concentrations simulating drought stress, stomatal closure intensified and stomatal density decreased in both groups (Figure 4F,G). Notably, overexpressed strains exhibited a greater degree of stomatal closure and a lower stomatal density relative to natural-type plants, which likely reduces transpirational water loss and contributes to improved drought adaptation.

### 2.5. Omics Analysis Under ABA Stress

Natural-type *P. davidiana × P. bolleana* (#NT) and overexpressed stranis (#OE) were subjected to ABA treatment and control (CK) conditions for 20 days. Each group consisted of three biological replicates, yielding a total of 12 leaf samples for transcriptome sequencing. Sequencing generated 80.34 Gb of high-quality clean data, with each sample producing over 6.12 Gb and a Q30 base percentage above 96.06%. Clean reads were aligned to the *Populus trichocarpa* reference genome, achieving alignment rates ranging from 65.6% to 70.83% across samples. Expression analysis identified 32,345 genes expressed in total, including 31,196 known genes and 1149 novel genes, as well as 63,946 transcripts, comprising 45,234 known and 18,712 novel transcripts.

Differential gene expression analysis was conducted using DESeq2 with thresholds set at |log_2_FC| ≥ 1 and *p* < 0.05 across four comparisons: CK#OE_vs_CK#NT, ABA#OE_vs_ABA#NT, ABA#NT_vs_CK#NT, and ABA#OE_vs_CK#OE. Gene expression exhibited significant dynamic changes under varying treatments. Under non-stress conditions, 671 differentially expressed genes (DEGs) were identified between the *PuWRKY22* overexpression (OE) and natural-type (NT) lines (CK#OE_vs_CK#NT) (Supplementary Figure S6), demonstrating that *PuWRKY22* overexpression significantly impacts basal transcriptional activity. ABA treatment markedly expanded transcriptional changes, with 1450 DEGs detected in the transgenic versus natural-type comparison under ABA stress (ABA#OE_vs_ABA#NT) (Supplementary Figure S6A). These findings suggest that ABA stress triggers extensive transcriptional reprogramming in *PuWRKY22*-overexpressed lines, highlighting the gene’s significant regulatory role in modulating the ABA signaling pathway.

To delineate the specific regulatory network of *PuWRKY22* under ABA stress, volcano plots were generated for DEGs in the ABA#OE_vs_ABA#NT and CK#OE_vs_CK#NT comparisons. Both comparisons revealed constitutive expression changes due to *PuWRKY22* overexpression, including genes such as *Ptr.BGL24* and *Ptr.WRKY22*, which were differentially expressed in both groups (Supplementary Figure S6C,D). Notably, *Ptr.WRKY22*, the homolog of *PuWRKY22*, exhibited the most significant upregulation in the ABA#OE_vs_ABA#NT group, indicating strong induction by ABA treatment. Additionally, *Ptr.PYL4* (Potri.006G104100.v4.1), a core ABA receptor gene within the ABA signaling pathway, was significantly upregulated, suggesting a central role for *PuWRKY22* in ABA signal modulation. To isolate genes regulated specifically by *PuWRKY22* in response to ABA, independent of constitutive effects, Venn diagram analysis identified 1312 unique DEGs exclusive to the ABA#OE_vs_ABA#NT comparison. These genes represent candidate targets regulated by *PuWRKY22* under ABA stress (Supplementary Table S1) (Supplementary Figure S6B).

**Figure 5:**
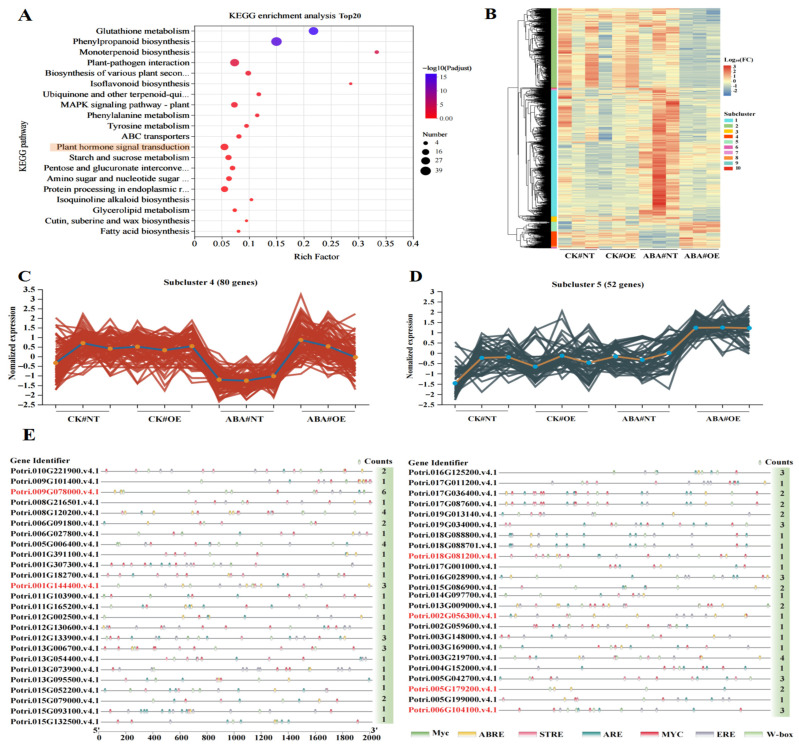
Functional enrichment, expression patterns, and promoter cis-element analysis of genes specifically regulated by *PuWRKY22* under ABA stress: (**A**) KEGG pathway enrichment analysis of 1312 genes uniquely regulated by *PuWRKY22* under ABA treatment. (**B**) Overall expression pattern clustering of the 1312 genes. (**C**) Detailed expression pattern analysis of Subcluster 4 (80 genes). (**D**) Detailed expression pattern analysis of Subcluster 5 (52 genes). (**E**) Visualization of promoter regions containing W-box cis-acting elements within genes from Subclusters 4 and 5. The genes marked in red are all key research areas and have been described in the previous results section.

KEGG enrichment analysis of the 1312 *PuWRKY22*-regulated genes under ABA stress revealed significant enrichment in glutathione metabolism and phenylpropanoid biosynthesis pathways, which are primarily associated with metabolic processes (Figure 5A) (Supplementary Table S2). However, as the core hormone mediating stress responses, ABA signal transduction represents a more direct and functionally relevant pathway for *PuWRKY22*’s role as a transcription factor. Among the 22 genes enriched in the plant hormone signal transduction pathway were key regulators such as *Ptr.PYL4* (Potri.006G104100.v4.1), *Ptr.ARF3* (Potri.011G059300.v4.1), *Ptr.GH3.6* (Potri.013G151100), *Ptr.TGA10* (Potri.016G049200), *Ptr.DELLA* (Potri.017G018600), and *Ptr.ERF1B* (Potri.010G072300). Notably, *Ptr.PYL4, Ptr.ARF3*, and *Ptr.GH3.6* showed significant upregulation in the ABA#OE_vs_ABA#NT comparison (Figure 5A), suggesting that *PuWRKY22* may directly or indirectly regulate multiple components within the plant hormone signaling network.

Expression pattern analysis of the 1312 *PuWRKY22*-dependent, ABA-stress-regulated target genes identified two key subclusters: Subcluster 4 (80 genes) and Subcluster 5 (52 genes), both exhibiting significantly higher expression levels in the ABA-treated transgenic lines (ABA#OE) compared to other groups (Figure 5B). These subclusters likely represent the molecular basis underlying the phenotypic advantages of the transgenic plants under ABA stress, making them prime candidates for further investigation. Within Subcluster 4, the average expression levels of the 80 genes showed no significant difference between non-stress natural-type (#NT) and ABA-treated transgenic (#OE) plants. However, under ABA stress, gene expression in the overexpressed line (#OE) was significantly elevated compared to both #NT and non-stressed plants, surpassing basal expression levels (Figure 5C). Representative genes in this group include *Ptr.PSB27-1* (Potri.002G056300.v4.1), *Ptr.APXT* (Potri.005G179200.v4.1), *Ptr.SOD2* (Potri.012G112301.v4.1), *Ptr.PYL4* (Potri.006G104100.v4.1), *Ptr.ABCB11* (Potri.010G003000.v4.1), and *Ptr.GH3.6* (Potri.013G151100.v4.1) (Supplementary Table S3). According to the change trend of the Subcluster 5 gene set, 52 genes only increased significantly when ABA coerced #OE, and there was no significant change in other treated group samples (Figure 5D), such as *Ptr.AAO1* (MSTRG.27576), *Ptr.ARF3* (Potri.011G059300.v4.1), *Ptr.NAC056* (Potri.011G123500.v4.1), *Ptr.MYBS1* (Potri.012G060300.v4.1), *Ptr.NYC1* (Potri.018G081200.v4.1), *Ptr.NAC71*(Potri.001G144400.v4.1), and other genes (Supplementary Table S4). The significantly higher expression of these genes in ABA#OE plants likely contributes to their enhanced phenotypic traits—including increased plant height and root length—and improved physiological parameters such as Fv/Fm and stomatal conductance compared to ABA-treated natural-type plants.

To further elucidate the regulatory relationships between *PuWRKY22* and the genes in Subclusters 4 (80 genes) and 5 (52 genes), this study analyzed the cis-acting elements within the 2000 bp promoter regions of all 132 genes. Visualization analysis identified that 49 of these genes contained W-box elements in their promoters (Figure 5E) (Supplementary Table S5). Notably, *Ptr.CHX24* (Potri.009G078000.v4.1) harbored six W-box elements; *Ptr.TKPR2* (Potri.008G120200.v4.1), *Ptr.PCMP-H69* (Potri.005G006400.v4.1), and *Ptr.PPH* (Potri.003G219700.v4.1) each contained four W-box elements; and *Ptr.NAC071* (Potri.001G144400.v4.1), *Ptr.PYL4* (Potri.006G104100.v4.1), and several other genes contained three W-box elements. Generally, a higher number of W-box elements within a promoter region correlates with stronger enrichment of WRKY proteins and greater likelihood of binding by the *PuWRKY22* transcription factor.

**Figure 6:**
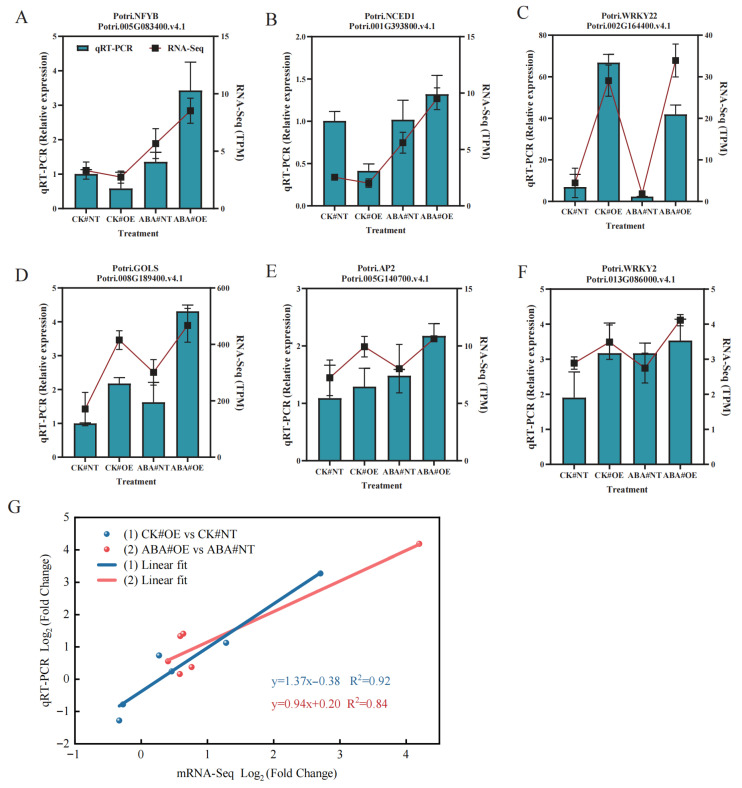
Validation analysis of RNA-Seq data by qRT-PCR. (**A**–**F**) Correlation analysis of expression levels for six randomly selected mRNAs measured by RNA-Seq (TPM normalized) and qRT-PCR (relative expression normalized to reference genes). (**G**) Linear regression plot of RNA-Seq log_2_ (FC) versus qRT-PCR log_2_ (FC) for the CK#OE vs. CK#NT and ABA#OE vs. ABA#NT comparisons. Note: Data are presented as mean ± SD.

To validate the differential expression results, qRT-PCR analysis was conducted on six randomly selected mRNAs using RNA samples from 12 libraries (three biological replicates for each of the four treatment groups). The qRT-PCR expression patterns were consistent with the RNA-Seq data, confirming the reliability of the transcriptomic analysis (Figure 6A–F). Figure 6G displays linear regression plots of RNA-Seq log_2_ (FC) versus qRT-PCR log_2_ (FC) for the CK#OE vs. CK#NT and ABA#OE vs. ABA#NT comparisons. Adjusted R^2^ values all exceeded 0.8, demonstrating strong concordance between RNA-Seq and qPCR log_2_ fold changes, which validates the reliability of the transcriptomic data.

**Figure 7:**
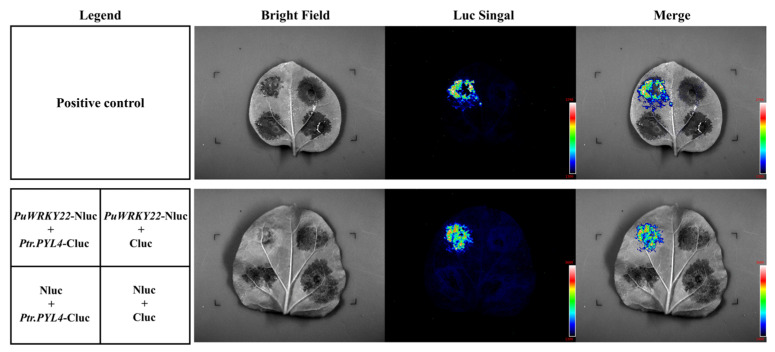
Validation diagram of luciferase complementation of PuWRKY22 protein and Ptr. PYL4 protein (Legend: schematic diagram of the location of Agrobacterium combination injection tobacco; bright field: bright field; Luc signal: fluorescent signal; merge: superposition of brightfield image and fluorescent signal image).

To investigate whether the PuWRKY22 protein interacts with Ptr.PYL4, a luciferase complementation assay was performed in tobacco leaves. The detection of a luciferase luminescence signal (Figure 7) confirmed the interaction between PuWRKY22 and Ptr.PYL4 proteins.

**Figure 8:**
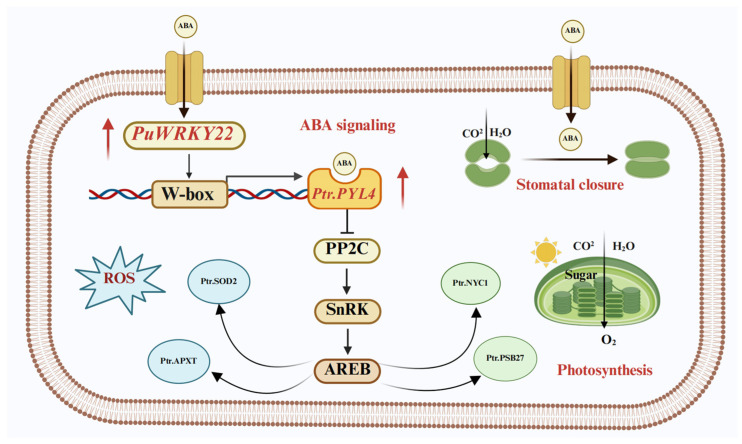
Schematic diagram illustrating the molecular mechanism of *PuWRKY22*-mediated regulation under ABA stress. *PuWRKY22* directly binds to W-box elements in the promoter of *Ptr.PYL4*, enhancing its transcription and promoting stomatal closure to reduce water loss. Concurrently, *PuWRKY22* activates ABA-responsive genes involved in photosynthesis (e.g., *Ptr.NYC1*, *Ptr.PSB27-1*) and ROS scavenging (e.g., *Ptr.SOD2*, *Ptr.APXT*) through the ABRE-mediated ABA signaling pathway, thereby improving drought tolerance. Indirect regulation of genes lacking W-box elements, such as *Ptr.ARF3* and *Ptr.GH3.6*, likely balances stress responses with growth. This integrated regulatory network enhances plant adaptation to drought stress.

Combining the KEGG enrichment analysis of the plant hormone signal transduction pathway with promoter cis-element visualization of genes in Subclusters 4 (80 genes) and 5 (52 genes), it is speculated that *PuWRKY22* preferentially binds to the promoter of *Ptr.PYL4*, enhancing its transcription. This likely accelerates stomatal closure in overexpressing plants, reduces stomatal density, and thereby decreases water loss, positioning *Ptr.PYL4* as a key target in elucidating how *PuWRKY22* integrates ABA signaling. Additionally, photosynthesis-related genes such as *Ptr.NYC1* (Potri.018G081200.v4.1) and *Ptr.PSB27-1* (Potri.002G056300.v4.1), along with ROS scavenging genes *Ptr.SOD2* (Potri.012G112301.v4.1) and *Ptr.APXT* (Potri.005G179200.v4.1), all contain ABRE cis-acting elements regulated by ABF transcription factors in the ABA pathway activated downstream of *PuWRKY22*, thereby enhancing drought tolerance in the overexpressing plants (Figure 8). However, the promoters of *Ptr.ARF3* and *Ptr.GH3.6*—which are also involved in hormone signaling—lack W-box elements, suggesting that *PuWRKY22* may regulate their expression indirectly to balance stress adaptation with growth maintenance in transgenic plants.

## 3. Discussion

This study systematically elucidated the biological function and molecular regulatory mechanisms of the *PuWRKY22* gene in *P. ussuriensis* under drought stress, highlighting its promising potential for molecular breeding aimed at enhancing drought tolerance in woody plants.

This study first obtained transcriptomic data from *P. trichocarpa* leaves treated with various hormones for 3 h. Differential gene expression analysis identified the *PtrWRKY22* gene (ID: Potr.002G164400) as strongly responsive to ABA signaling. Using homologous cloning methods, the corresponding homolog *PuWRKY22* was successfully isolated from *P. ussuriensis*. qRT-PCR analysis showed that *PuWRKY22* exhibits significant tissue-specific expression and stress-responsive characteristics, including involvement in ABA signaling pathways. As a key drought response signal in plants, ABA activates specific WRKY transcription factors [21]. These transcription factors regulate downstream response genes by binding to specific DNA sequences, thereby enhancing plant drought tolerance through mechanisms such as controlling stomatal closure [22], promoting root development [23], and increasing the synthesis of osmotic regulatory substances [24]. For instance, *ItfWRKY70* improves drought tolerance by elevating ABA levels, modulating stomatal aperture, and activating the reactive oxygen species (ROS) scavenging system, playing a positive role in drought resistance [25]. ABA-responsive cis-acting elements (ABREs) are key components in plant cell responses to ABA signals [26]. Promoter analysis revealed that *PuWRKY22* contains multiple ABRE elements, supporting its involvement in drought resistance through ABA-dependent pathways. Promoter analysis revealed that *PuWRKY22* contains multiple ABA-responsive cis-acting elements (ABREs), with three ABRE motifs enriched in its promoter region. These findings support the role of *PuWRKY22* in drought resistance via ABA-dependent signaling pathways, similar to the soybean *WRKY20* gene [27]. To further validate the drought tolerance function of *PuWRKY22*, transgenic tobacco and *P. davidiana × P. bolleana* lines overexpressing the gene were treated with ABA and sorbitol to simulate drought stress [28]. Combining phenotypic observations and physiological measurements, an in-depth functional assessment was conducted for this study. The overexpression of *PuWRKY22* significantly enhanced drought tolerance in both species. During germination, transgenic plants exhibited higher germination rates, increased vigor, and greater green leaf percentages, paralleling the drought tolerance mechanism of *SbWRKY45* [29]. In the vegetative growth stage, *PuWRKY22* promoted root development and maintained photosynthetic efficiency, thereby improving water uptake and photosystem stability. Further physiological analyses revealed that overexpressed lines had more efficient reactive oxygen species (ROS) scavenging systems and enhanced membrane protection, suggesting that *PuWRKY22* confers drought resistance through multiple regulatory pathways.

Luciferase complementation assays demonstrated that the WRKY domain of *PuWRKY22* specifically binds to W-box elements, suggesting that *PuWRKY22* regulates drought stress through a mechanism similar to that of *SbWRKY30*. Both transcription factors induce drought-responsive gene expression by binding to W-box motifs in the promoters of target genes, thereby enhancing plant drought resistance [30]. *PuWRKY22* functions as a key downstream transcription factor in the ABA signaling pathway. It is induced by core ABA signaling components (PYL-PP2C-SnRK-AREB) and directly regulates antioxidant and cell-wall-related genes such as *Ptr.SOD2* and *Ptr.APXT.* Additionally, *PuWRKY22* may influence chlorophyll metabolism and photosynthesis-related genes, including *Ptr.NYC1* [31] and *Ptr.PSB27* [32]. Through these multifaceted roles, *PuWRKY22* contributes to abiotic stress responses linked to ABA signaling—enhancing ROS scavenging capacity, maintaining cellular integrity, and regulating energy metabolism. These findings provide a vital theoretical basis for understanding the molecular mechanisms of stress resistance in *P. ussuriensis* and for leveraging *PuWRKY22* in molecular breeding for improved drought tolerance.

This study has several limitations, including an incomplete characterization of the downstream target genes regulated by *PuWRKY22* and the detailed mechanisms of their interactions. Additionally, experiments involving the seedling stage of *P. davidiana × P. bolleana* and the generation of *PuWRKY22* knockout plants were not conducted. Future research should integrate techniques such as ChIP-seq [33] and EMSA [34] to further validate the binding specificity of *PuWRKY22* to W-box elements. In summary, *PuWRKY22* enhances drought resistance in woody plants through multiple molecular pathways, providing a foundation for further exploration of its biological functions and mechanisms in drought stress responses in *P. ussuriensis*.

## 4. Materials and Methods

### 4.1. Plant Growth and Treatment

This study utilized tissue-cultured seedlings of natural-type *P. ussuriensis* and *P. davidiana × P. bolleana* provided by the Key Laboratory of Saline-Alkali Soil Research, Northeast Forestry University. Additionally, T3-generation transgenic tobacco (*Nicotiana tabacum*) lines overexpressing the *PuWRKY22* gene, generated via genetic transformation and preserved in the same laboratory, were employed. All plants were grown under controlled conditions at 25 °C. The photoperiod was 16 h of light and 8 h of darkness. To examine the spatiotemporal expression patterns of *PuWRKY22*, roots, stems, and leaves of *P. ussuriensis* were collected for gene expression analysis across different tissues. For stress response experiments, stem segments of *P. ussuriensis* were subjected to various abiotic stress treatments for 48 h, and changes in *PuWRKY22* expression were monitored. To analyze the temporal expression profile under osmotic stress, *P. ussuriensis* leaves were harvested at 0, 6, 12, 24, and 48 h, respectively, following treatment with 200 mM sorbitol. For stress resistance assays in transgenic tobacco, plants were grown on media supplemented with varying concentrations of sorbitol (0, 200, 250, and 300 mM) and ABA (0, 2.5, 5.0, and 7.5 μM) (Fangcheng Biotechnology, Beijing, China). In *P. davidiana × P.bolleana*, drought and ABA responses were evaluated under treatment with 300 mM sorbitol and 7.5 μM ABA. For transcriptomic analyses, *P. davidiana × P.bolleana* plants were treated with 7.5 μM ABA to induce stress-responsive gene expression.

### 4.2. RNA and DNA Extraction and Gene Cloning

Total RNA was extracted from plant tissues using TRIzol reagent (Vazyme, Nanjing, China), following the manufacturer’s protocol [35], and subsequently reverse-transcribed into complementary DNA (cDNA). Genomic DNA was extracted using the cetyltrimethylammonium bromide (CTAB) method [36]. The coding sequence (CDS) of *PtrWRKY22* (Gene ID: *Potr.002G164400*) was retrieved from the *Populus trichocarpa v4.1* genome database via Phytozome v13.1 (https://phytozome-next.jgi.doe.gov/ (accessed on 15 September 2021)). Based on this sequence, gene-specific primers *PuWRKY22-F1/R1* (listed in Supplementary Table S6) were designed using Primer 5.0 software, targeting regions flanking the 5′ and 3′ untranslated regions (UTRs) of the coding sequence. Using cDNA synthesized from *P. ussuriensis* as the template, the *PuWRKY22* gene was amplified by reverse transcription PCR (RT-PCR) [37] for subsequent cloning and downstream functional analysis.

### 4.3. Analysis of the Expression Specificity of the PuWRKY22 Gene

Tissue-cultured seedlings of *P. ussuriensis* were subcultured for four weeks under controlled conditions. Total RNA was then extracted from stems, roots, and leaves, followed by reverse transcription to synthesize cDNA. Gene-specific primers for *PuWRKY22* and the housekeeping gene *PuActin* (used as an internal reference) were designed using Primer 5.0 software (sequences listed in Supplementary Table S6). Quantitative real-time PCR (qRT-PCR) was performed using an Agilent Mx3000P real-time PCR system (Agilent, USA). Relative expression levels were calculated using the 2^−ΔΔCt^ method: Fold Change = 2^−ΔΔCt^ = 2^−[(Ct(target gene GOI, test) − Ct(internal reference gene REF, test)) − (Ct(GOI, control) − Ct(REF, control))]. Results were visualized and statistically analyzed using GraphPad Prism 9.0 software.

### 4.4. Subcellular Localization of PuWRKY22 Protein

The subcellular localization of the PuWRKY22 protein was first predicted using the Plant-mPLoc online tool. To experimentally validate the localization, the full-length coding sequence of *PuWRKY22* (without the stop codon) was cloned into the pGWB5 vector downstream of the CaMV 35S promoter to generate a GFP fusion construct (pGWB5-35S-*PuWRKY22*-GFP). The recombinant plasmid was introduced into the *Agrobacterium tumefaciens* strain GV3101 (WEIDI, Shanghai, China) and subsequently infiltrated into tobacco leaves using an *Agrobacterium*-mediated transient expression method, as described previously [38]. GFP fluorescence signals were observed 48–72 h post-infiltration using a ZEISS Imager Z2 fluorescence microscope (ZEISS, Massachusetts, USA). The observed subcellular localization pattern was compared to the Plant-mPLoc prediction to confirm nuclear localization of the PuWRKY22 protein.

### 4.5. Construction and Activity Validation of the PuWRKY22 Gene Promoter

A 2000 bp sequence upstream of the start codon of the *PtrWRKY22* gene was selected as the putative promoter region. Cis-acting regulatory elements within this region were predicted using the PlantCARE online database. Gene-specific primers (Pro*PuWRKY22*-F1/R1, listed in Supplementary Table S6) were designed to amplify the promoter fragment. The amplified product was cloned into the pGWB3 vector upstream of the GUS reporter gene via LR recombination to generate the Pro*PuWRKY22*::GUS construct. The resulting recombinant plasmid was introduced into tobacco via *Agrobacterium*-mediated transformation to obtain transgenic tobacco plants. Tissues including roots, stems, leaves, and flowers from different developmental stages were collected from transgenic lines for GUS histochemical staining. Staining was performed according to the protocol described previously [39], and the expression patterns were observed and documented to evaluate spatial and temporal promoter activity (OLYMPUS SZX9, Tokyo, Japan).

### 4.6. Analysis of the Binding Characteristics of PuWRKY22 Protein and W-Box

To investigate the DNA-binding specificity of PuWRKY22, the full-length coding sequence was amplified using primers p62SK-*PuWRKY22*-F/R (Supplementary Table S6), which incorporated SalI and BamHI restriction sites and homologous arms. The amplified product was cloned into the pGreenII 62-SK vector. The vector was first digested with SalI and BamHI, and the linearized backbone was purified from agarose gel. Seamless cloning was performed at 37 °C for 30 min to ligate the *PuWRKY22* insert with the linearized vector, generating the recombinant construct pGreenII 62-SK-*PuWRKY22*. The ligation product was transformed into *Escherichia coli*, and positive clones were screened on kanamycin (Kana)-containing plates. The integrity of the recombinant plasmid was confirmed via dual enzyme digestion. To assess DNA-binding activity, synthetic cis-acting elements containing either the wild-type W-box (three tandem repeats of TTGAC(C/T)) or a mutated version (mW-box, three repeats of TAGACG) were prepared. Primers W-box-F/R (Supplementary Table S6) were used to amplify the corresponding fragments, which were purified from agarose gels. The effector plasmid pGreenII 62-SK-*PuWRKY22* and reporter constructs (pGreenII 0800-W-box-Luc, pGreenII 0800-mW-box-Luc, and the control pGreenII 0800-Luc) were co-transformed into *Agrobacterium tumefaciens* GV3101 (pSoup + p19) via electroporation [40]. *Agrobacterium* cultures were adjusted to OD_600_ = 1.5 and incubated at room temperature for 3 h. Equal volumes of the effector, reporter, and p19 strains were mixed at a 1:1 ratio and infiltrated into the abaxial side of four-week-old tobacco leaves. Following infiltration, the plants were kept in the dark for 12 h and then grown under standard conditions for 3 days. Subsequently, 100 μL of a D-luciferin-containing reaction buffer was injected into the infiltrated leaves and incubated for 7 min. Luciferase activity was detected using a high-sensitivity chemiluminescence imaging system (Tanon 4600SF, Tanon, Shanghai, China), allowing evaluation of the binding specificity of PuWRKY22 to the W-box and mW-box elements.

### 4.7. Genetic Transformation of PuWRKY22 and Detection of Physiological Indicators After Drought Treatment

#### 4.7.1. Tobacco Genetic Transformation and Identification

The recombinant plasmid harboring the *PuWRKY22* gene was introduced into the *Agrobacterium tumefaciens* strain EHA105 (WEIDI, Shanghai, China) via heat shock transformation. Verified *Agrobacterium* colonies were used to infect leaf discs of tobacco through *Agrobacterium*-mediated transformation. Infected explants were co-cultivated for 3 days on co-culture medium (½ MS + acetosyringone). Shoot regeneration was induced on selection medium supplemented with 50 mg/L hygromycin (Hyg). Successfully regenerated shoots were transferred to rooting medium (½ MS + 50 mg/L Hyg + 250 mg/L carbenicillin) to induce root formation and obtain stable transgenic lines. T3-generation transgenic tobacco lines and wild-type seeds were surface-sterilized and germinated on hygromycin-containing selection medium. After 20 days of growth, hygromycin-resistant seedlings were transplanted into soil and cultivated under controlled conditions. At the five-leaf, one-bud stage, genomic DNA was extracted from leaf tissue, and transgene integration was confirmed by PCR using *PuWRKY22*-F1/R1 primers (Supplementary Table S6). Quantitative real-time PCR (qRT-PCR) was subsequently performed to assess *PuWRKY22* expression levels in different transgenic lines. Lines with high expression levels (#4, #5, #6, and #7) were selected for further physiological and biochemical analyses.

#### 4.7.2. Genetic Transformation and Identification of *P. davidiana × P. bolleana*

*P. davidiana × P. bolleana* leaves were transformed using *Agrobacterium tumefaciens*-mediated infection. Following inoculation, the leaves were incubated in darkness for 3 days on co-culture medium (WPM supplemented with acetosyringone). After co-cultivation, explants were sterilized and transferred to selection medium (WPM + 0.5 mg/L 6-benzylaminopurine [6-BA] + 0.1 mg/L naphthaleneacetic acid [NAA] + 5 mg/L hygromycin [Hyg] + 250 mg/L carbenicillin [Carb]) with the adaxial surface facing upward, and cultured under a 16 h light/8 h dark photoperiod at 25 °C. Upon callus formation and shoot regeneration, the shoots were transferred to rooting medium (WPM + 5 mg/L Hyg + 250 mg/L Carb) to induce root development and generate transgenic *P. davidiana × P. bolleana* lines overexpressing *PuWRKY22*. Putative transgenic lines were confirmed by PCR using gene-specific primers. Quantitative real-time PCR (qRT-PCR) was subsequently conducted to assess *PuWRKY22* expression levels. Transgenic lines exhibiting high expression (#7, #11, and #12) were selected for downstream physiological and transcriptomic analyses.

#### 4.7.3. Drought Treatment

##### Genetically Modified Tobacco Strains Simulate Drought Treatment

The same batch of uniform and full T3-generation *PuWRKY22* genetically modified tobacco seeds (plant line #5, #6, #7) and wild-type (WT) seeds were selected, and after disinfection, they were chosen to contain different concentrations of sorbitol (0, 200, 250, 300 mm) or ABA (0, 2.5, 5, 7.5 μM) 1/2 MS culture medium (25 grains each/treatment) after 3 days of 4 °C springization and cultured horizontally at 25 °C (8/16 h light/dark), and statistics of germination potential, germination rate, and green leaf rate from seed sprouting were produced. In addition, WT and transgenic seeds were vertically cultured on 1/2 MS culture medium until the three-leaf stage, and the seedlings with the same growth were selected and transferred to 1/2 MS culture medium containing the same concentration of sorbitol or ABA. After 15 days of vertical incubation at 25 °C (8/16 h light/dark), the root length was measured (ImageJ), along with weight (analytical balance). At the same time, vertically cultivated T3-generation *PuWRKY22* genetically modified strains and WT strain seedlings (16 plants/groups) with the same growth were transplanted to the soil to ensure the same soil weight. They were placed at 25 °C (8/16 h light/dark) under the same conditions for 1 month. Seedlings with consistent growth conditions were selected for natural drought treatment, and the phenotypic changes at 0 days, 15 days of drought, and 3 days of rewatering were dynamically recorded. The same amount of water (100 ml) was used during recovery, and the open chlorophyl fluorescence imaging system (FluorCam 1300, Czech PSI, Czech, Europe) was used to capture Fv/Fm images, with three repetitions per group.

##### The Overexpressed *P. davidiana × P. bolleana* Strain Simulated Drought Treatment

Natural-type (NT) and overexpressed *P. davidiana × P. bolleana* strain culture seedlings (#7, #11 and #12) were chosen and allowed to grow for about 3 weeks. Cut stem segments with 1-3 leaf lengths at the top and insert them into WPM rooting medium, 300 mM Sorbitol and 7.5 μM ABA stress medium respectively. Twenty days later, the growth status and phenotypic changes were observed. Leaves of the natural-type and overexpressed *P. davidiana × P. bolleana* strain under normal culture and stress culture conditions were collected, respectively, for phenotypic determination, with three replicates in each group.

#### 4.7.4. Measurement of Physiological Indicators

##### Determination of Leaf Electrolyte Permeability

Leaf samples were rinsed 2–3 times with ultrapure water to remove surface contaminants and then cut into small uniform pieces and immersed in 15 mL of ultrapure water in test tubes. Tubes containing only ultrapure water served as blank controls. The samples were incubated on a shaker at 40 rpm for 90 min at room temperature, after which the initial conductivity (C_1_) of the sample solution and control (CK_1_) was measured using a DDS-307 conductivity meter (DDS-307, Shanghai, China). Subsequently, the samples were boiled in a 100 °C water bath for 10 min to release total electrolytes and cooled to room temperature, and the final conductivity (C_2_, control CK_2_) was measured. Relative electrolyte leakage (EL%) was calculated using the formula EL% = [(C_1_ − CK_1_)/(C_2_ − CK_2_)] × 100%. This measurement reflects membrane integrity and cellular damage under stress conditions.

##### Determination of Chlorophyll Content and Fluorescence Parameters

To evaluate the effects of drought stress on photosynthetic performance, both *PuWRKY22*-transgenic and wild-type plants were analyzed for chlorophyll content and fluorescence parameters. Chlorophyll content was measured using a SPAD-502PLUS chlorophyll meter (Konica Minolta, Tokyo, Japan). For each plant, representative leaves from the same developmental stage and position were selected. Three anatomical locations per leaf were designated as fixed measurement points, and each point was measured three times. The average of these values was recorded as the chlorophyll content (SPAD value) for that sample. Under natural drought conditions, overall plant growth was visually assessed, and phenotypic images were recorded to document morphological responses. To assess the photochemical efficiency of photosystem II (PSII), the FluorCam fluorescence imaging system was employed. The maximum quantum yield of PSII (Fv/Fm) was measured in dark-adapted leaves. Fluorescence images were captured, and all datasets were analyzed using a standardized color scale range of 0.2–0.8 to ensure consistency and comparability across samples.

##### Determination of Malondialdehyde (MDA) Content

A total of 0.2 g of fresh leaves was taken from a main vein, and 3 mL of 100 mM phosphate-buffered saline was added (PBS pH7.8). Then, it was ground on ice into a homogenate, transferred to a centrifuge tube, and centrifuged at 4 °C and 12,000 rpm for 20 min. The supernatant was the enzyme solution. A total of 100 μL of the enzyme solution of the sample was taken to be tested and added to a 1.5 mL centrifuge tube. Then, 1 mL of 0.25% thiobarbituric acid (TBA) solution was added. At the same time, the control tube was set up (the enzyme solution was replaced with 100 μL of 100 mM PBS pH 7.8). The reaction mixture was placed in a boiling water bath for 15 min, and then cooled on ice for 5 min. A total of 200 μL of the cooled reaction mixture was taken, and its absorbance values were measured at 532 nm and 600 nm (Tecan M200 PRO, Switzerland, Europe). The following formula was used for calculations: malondialdehyde content (nmol/g FW) = (A532 − A600) × Vr × (V/Vt)/155 × 1000/W. In the formula, A532 represents the absorbance at 532 nm, A600 represents the absorbance at 600 nm, Vr is the volume of the reaction mixture, V is the total volume of the crude enzyme solution, Vt is the volume of the crude enzyme solution used in the detection tube, and W is the fresh weight of the sample (g). The extinction coefficient of MDA-TBA at 532 nm is 155.

##### DAB and NBT Staining

3,3′-Diaminobenzidine (DAB) and nitro blue tetrazolium (NBT) staining were performed to visualize reactive oxygen species (ROS) accumulation in plant leaves. DAB staining was used to detect hydrogen peroxide (H_2_O_2_) accumulation [41], while NBT staining was employed to assess superoxide anion (O_2_^−^) production in response to drought stress [42]. Leaves were collected from the same developmental position of both well-watered and drought-stressed plants, labeled, and placed into 50 mL centrifuge tubes containing either 0.1% (*w*/*v*) DAB solution (pH 3.8) or 0.5 mg/mL NBT solution. Samples were incubated at 37 °C in the dark for 12–16 h, followed by light exposure for 2 h to enhance signal development. After staining, leaves were decolorized in 95% ethanol with multiple changes until chlorophyll was completely removed, ensuring clear visualization of ROS staining. Images of DAB-stained leaves were captured using an HP flatbed scanner for comparative analysis (Tecan M200 PRO, Switzerland, Europe).

### 4.8. Stomata Structure Analysis

Leaf samples were cut into approximately 2 × 5 mm strips using a sterile blade and immediately immersed in a 2.5% (*v*/*v*) glutaraldehyde/formaldehyde fixative solution (pH 6.8). The samples were subjected to vacuum infiltration until they were fully submerged and suspended in the fixative and then stored at 4 °C for at least 1.5 h to ensure adequate fixation. Following fixation, samples were rinsed twice with 0.1 mol/L phosphate buffer (pH 6.8) to remove excess fixative. A graded ethanol series (50%, 70%, 90%) was used for dehydration, followed by two rinses in 100% ethanol. The ethanol was then replaced with tert-butanol for 15 min to aid drying. Samples were dried for 4 h and then mounted on aluminum stubs with the epidermal surface facing upward. Finally, samples were sputter-coated (if applicable) and examined using a tungsten filament scanning electron microscope (Hitachi S-3400N, Tokyo, Japan). Images were acquired to analyze stomatal morphology and pore structure under various experimental conditions.

### 4.9. Bioinformatics Analysis

Transcriptome sequencing was performed on leaf samples collected from natural-type *P. davidiana × P. bolleana* plants (NT) and *PuWRKY22*-transgenic lines (OE#7,#11, and #12) under both control (CK) and ABA treatment conditions (20 days post-treatment). Quality control of raw sequencing reads was assessed by analyzing base composition (A/T/G/C), base quality score distributions (Phred scores), and base error rates. Low-quality reads and adapter sequences were filtered using fastp (https://github.com/OpenGene/fastp (accessed on 3 March 2025)). Clean reads were then aligned to the *Populus trichocarpa* v4.1 reference genome (chosen as the model genome for the *Populus* genus) using HiSat2 (http://ccb.jhu.edu/software/hisat2/index.shtml (accessed on 4 March 2025)). Gene and transcript expression levels were quantified using RSEM (http://deweylab.github.io/RSEM/ (accessed on 4 March 2025)), yielding raw read counts and normalized transcript abundance in TPM (Transcripts Per Million). Differentially expressed genes (DEGs) were identified using DESeq2 (http://bioconductor.org/packages/stats/bioc/DESeq2/ (accessed on 5 March 2025)), with thresholds set at |log_2_ (Fold Change)| ≥ 1 and FDR-adjusted *p*-value < 0.05. To functionally annotate DEGs, KEGG pathway enrichment analysis was performed (https://www.genome.jp/kegg (accessed on 5 March 2025)). The upstream 2000 bp regions from the transcription start sites of selected genes were retrieved via the Ensembl Plants database (http://plants.ensembl.org/Populus_trichocarpa/Info/Index (accessed on 6 March 2025)) and analyzed for cis-acting regulatory elements using PlantCARE (https://bioinformatics.psb.ugent.be/webtools/plantcare/html/ (accessed on 6 March 2025)). The distribution of regulatory elements was visualized using the “Visualize Motif in Sequence” function in TBtools v2.025. To validate transcriptomic results, six DEGs were randomly selected for qRT-PCR validation. Gene-specific primers (product size 100–150 bp, Tm 57 ± 2 °C) were designed using Primer-BLAST (https://www.ncbi.nlm.nih.gov/tools/primer-blast/ (accessed on 20 March 2025)). First-strand cDNA was synthesized from 1 μg of total RNA using reverse transcriptase. The qRT-PCR reaction system (20 μL total volume) consisted of 10 μL SYBR Green Master Mix, 1 μL cDNA template, 1 μL each of forward and reverse primers, and 7 μL ddH_2_O. Relative expression levels were calculated using the 2^−ΔΔCt^ method.

## 5. Conclusions

This study successfully cloned the *PuWRKY22* gene from *P. ussuriensis*, encoding a nuclear-localized protein induced by ABA and various abiotic stresses. Drought stress experiments in transgenic tobacco and *P. davidiana × P.bolleana* demonstrated that the overexpression of *PuWRKY22* significantly enhanced seed germination rates, root length, and biomass accumulation, while reducing ROS levels and membrane lipid peroxidation. Luciferase complementation assays confirmed that the *PuWRKY22* transcription factor binds specifically to W-box cis-acting elements, suggesting its role in regulating gene expression. Stomatal structure analysis revealed that transgenic plants exhibited more pronounced stomatal closure under drought conditions, effectively reducing water loss through transpiration. Transcriptome analysis further showed that *PuWRKY22* activates downstream stress response pathways by upregulating the ABA receptor gene *Ptr.PYL4* (*Potri.006G104100.v4.1*). Collectively, this study elucidates, for the first time, the molecular mechanism by which *PuWRKY22* regulates drought responses in woody plants via the ABA signaling pathway and protein interactions, providing valuable genetic resources for improving drought tolerance in poplars.

## Data Availability

Data will be made available on request.

## Supplementary Materials

The following supporting information can be downloaded at: https://www.mdpi.com/article/10.3390/plants14172621/s1, Supplementary Figure S1 Differential gene expression heat map of *Populus trichocarpa* leaves treated with different hormones for 3 h. Supplementary Figure S2 Expression specificity analysis of the *PuWRKY22* gene. A: Expression characteristics of PuWRKY22 gene in different tissues and organs; B: Changes in the expression of *PuWRKY22* gene in leaves of *P. ussuriensis* Kom under 200 mM Sorbitol stress; C: Expression of *PuWRKY22* in the stems of *P. ussuriensis* Kom at 48 h under six different stress treatments; Note: Data are presented as mean ± SD; Bars labeled with different letters differ significantly (one-way ANOVA, Tukey’s HSD test, *p* < 0.05). Supplementary Figure S3 Transgenic *PuWRKY22* Tobacco Phenotypes at Germination Stage under ABA stress (0, 2.5, 5, and 7.5 μM). A: Phenotypes under varying ABA stress concentrations; B: Germination potential; C: Germination rate; D: Green leaf rate; Note: Data are presented as mean ± SD; Bars labeled with different letters differ significantly (one-way ANOVA, Tukey’s HSD test, *p* < 0.05). Supplementary Figure S4 Transgenic *PuWRKY22* Tobacco Phenotypes at Germination Stage under Sorbitol stress (0, 200, 250, and 300 mM). A: Phenotypes under varying Sorbitol stress concentrations; B: Germination potential; C: Germination rate; D: Green leaf rate; Note: Data are presented as mean ± SD; Bars labeled with different letters differ significantly (one-way ANOVA, Tukey’s HSD test, *p* < 0.05). Supplementary Figure S5 Phenotypic and Physiological Responses of Transgenic PuWRKY22 Tobacco at the Three-Leaf Stage under ABA Stress. A: Phenotype of transgenic *PuWRKY22* tobacco lines; B: Determination of fresh weight of transgenic *PuWRKY22* tobacco lines; C: Determination of root length of transgenic *PuWRKY22* tobacco lines; Note: Data are presented as mean ± SD; Bars labeled with different letters differ significantly (one-way ANOVA, Tukey’s HSD test, *p* < 0.05). Supplementary Figure S6 Phenotypic and physiological responses of transgenic *PuWRKY22* tobacco at the Three-Leaf Stage under Sorbitol stress. A: Phenotype of transgenic *PuWRKY22* tobacco lines; B: Determination of fresh weight of transgenic *PuWRKY22* tobacco lines; C: Determination of root length of transgenic *PuWRKY22* tobacco lines; Note: Data are presented as mean ± SD; Bars labeled with different letters differ significantly (one-way ANOVA, Tukey’s HSD test, *p* < 0.05). Supplementary Figure S7. Identification of PuWRKY22-specific regulatory targets under ABA stress. A: Number of differentially expressed genes (DEGs) in comparisons CK#OE_vs_CK#NT and ABA#OE_vs_ABA#NT; B: Venn diagram of DEGs between CK#OE_vs_CK#NT and ABA#OE_vs_ABA#NT; C: Volcano plot of DEGs in CK#OE_vs_CK#NT; D: Volcano plot of DEGs in ABA#OE_vs_ABA#NT. Supplementary Table S1: 1312 candidate target genes specifically responsive to ABA stress regulated by PuWRKY22. Supplementary Table S2: KEGG analysis of candidate target genes regulated by PuWRKY22 in response to ABA stress. Supplementary Table S3: Gene annotation information for Subcluster 4. Supplementary Table S4: Gene annotation information for Subcluster 5. Supplementary Table S5: Promoter prediction of the W-bow element in the Subcluster 4 and Subcluster 5 genes. Supplementary Table S6: Primer sequence summary.
