# Supplementary material for: Populus ussuriensis PuWRKY22 Transcription Factor Activates the ABA Receptor PYL4 to Enhance Drought Resistance
Source: Plants (Basel). 2025 Aug 23;14(17):2621. doi: 10.3390/plants14172621 (PMC12430080; doi:10.3390/plants14172621)
Supplement: Supplementary file 1 [file plants-14-02621-s001.zip › Supplementary materials .pdf]

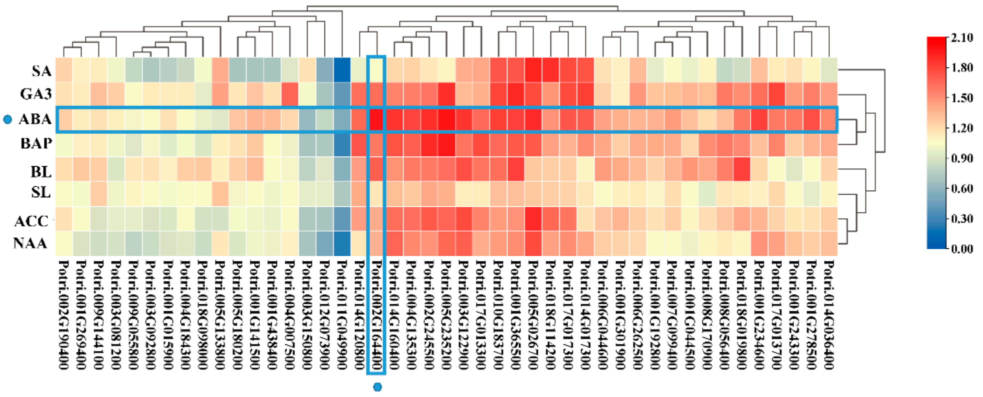

Supplementary Fig.S1 Differential gene expression heat map of *Populus trichocarpa* leaves treated with different hormones for 3 hours.

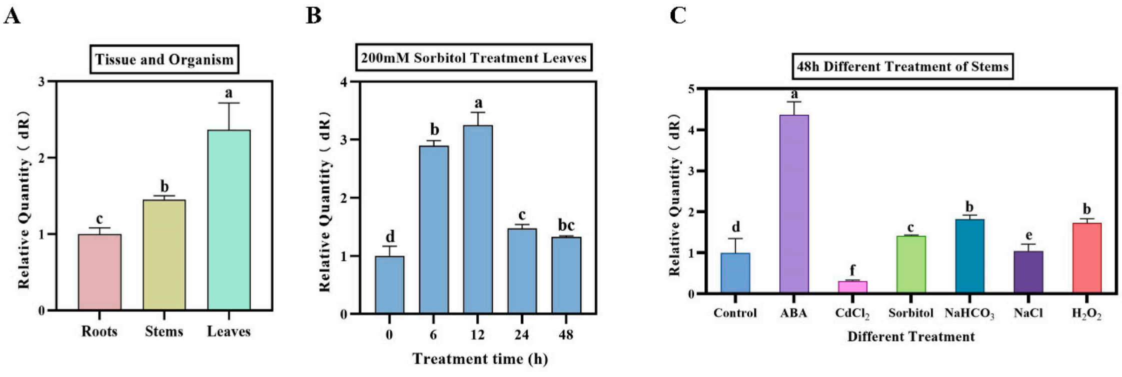

Supplementary Fig.S2 Expression specificity analysis of the *PuWRKY22* gene. A: Expression characteristics of PuWRKY22 gene in different tissues and organs; B: Changes in the expression of *PuWRKY22* gene in leaves of *P. ussuriensis* Kom under 200 mM Sorbitol stress; C: Expression of *PuWRKY22* in the stems of *P. ussuriensis* Kom at 48 h under six different stress treatments; Note: Data are presented as mean ± SD; Bars labeled with different letters differ significantly (one-way ANOVA, Tukey's HSD test, $p < 0.05$).

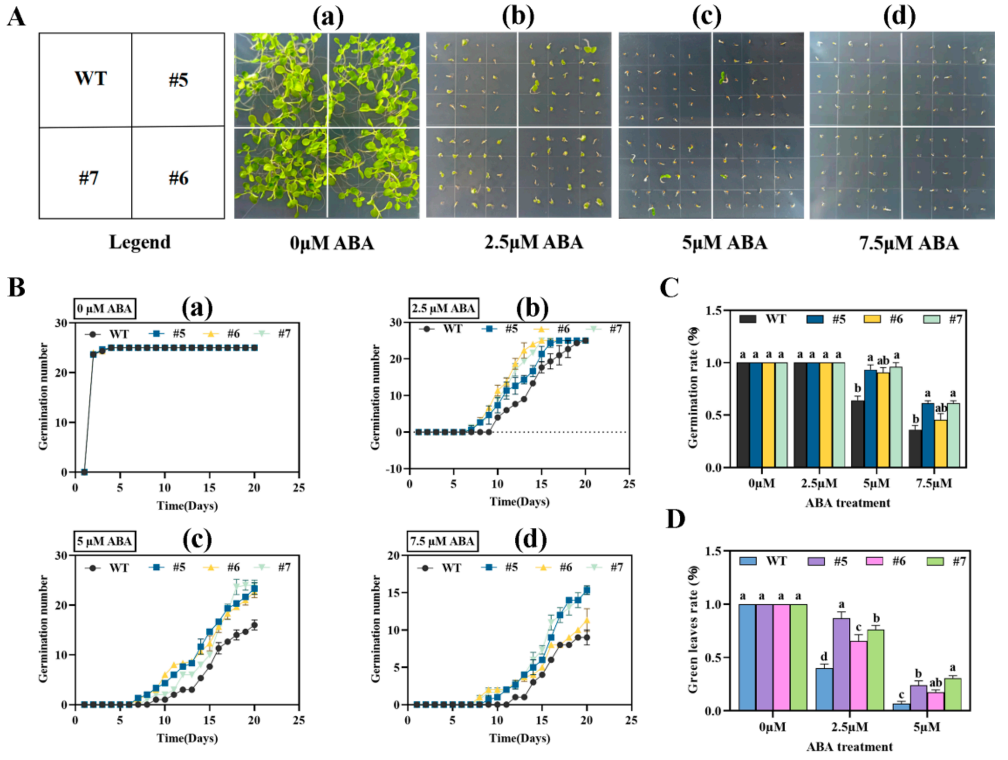

Supplementary Fig.S3 Transgenic *PuWRKY22* Tobacco Phenotypes at Germination Stage under ABA stress (0, 2.5, 5, and 7.5 μM). A: Phenotypes under varying ABA stress concentrations; B: Germination potential; C: Germination rate; D: Green leaf rate; Note: Data are presented as mean ± SD; Bars labeled with different letters differ significantly (one-way ANOVA, Tukey's HSD test, $p < 0.05$).

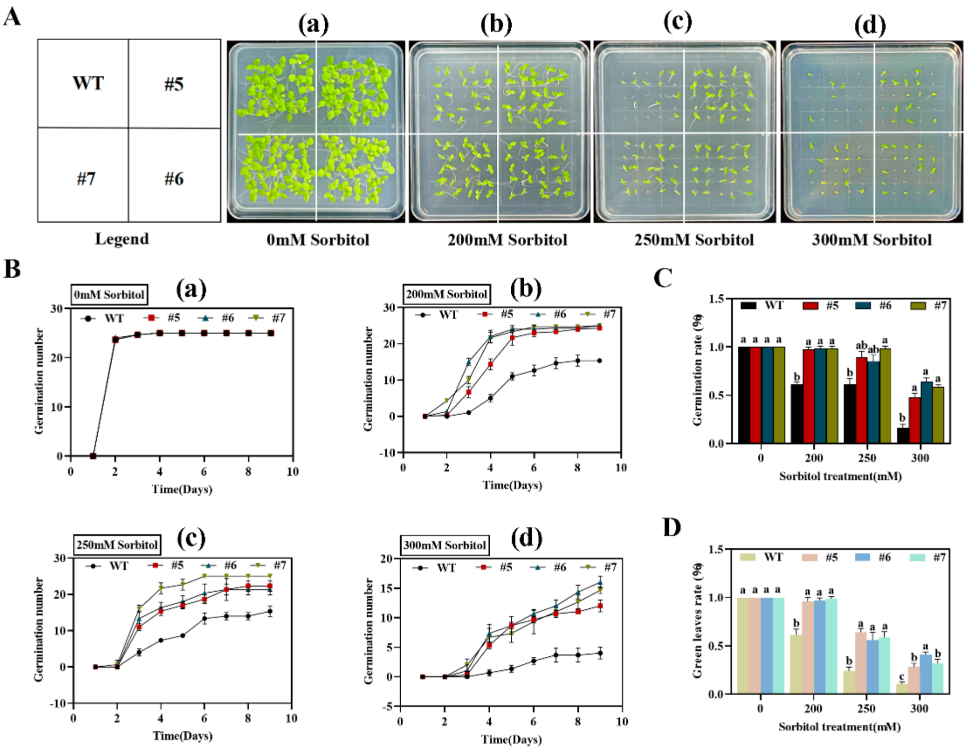

Supplementary Fig.S4 Transgenic *PuWRKY22* Tobacco Phenotypes at Germination Stage under Sorbitol stress (0, 200, 250, and 300 mM). A: Phenotypes under varying Sorbitol stress concentrations; B: Germination potential; C: Germination rate; D: Green leaf rate; Note: Data are presented as mean ± SD; Bars labeled with different letters differ significantly (one-way ANOVA, Tukey's HSD test, $p < 0.05$).

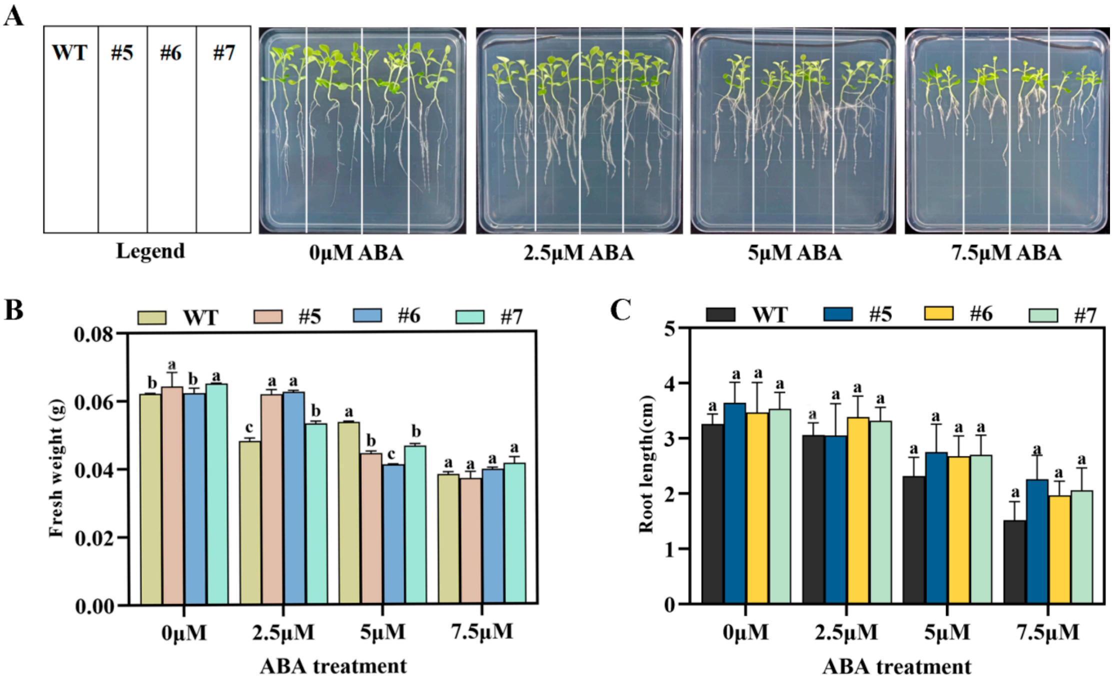

Supplementary Fig.S5 Phenotypic and Physiological Responses of Transgenic PuWRKY22 Tobacco at the Three-Leaf Stage under ABA Stress. A: Phenotype of transgenic *PuWRKY22* tobacco lines; B: Determination of fresh weight of transgenic *PuWRKY22* tobacco lines ; C: Determination of root length of transgenic *PuWRKY22* tobacco lines ; Note: Data are presented as mean ± SD; Bars labeled with different letters differ significantly (one-way ANOVA, Tukey’s HSD test, $p < 0.05$).

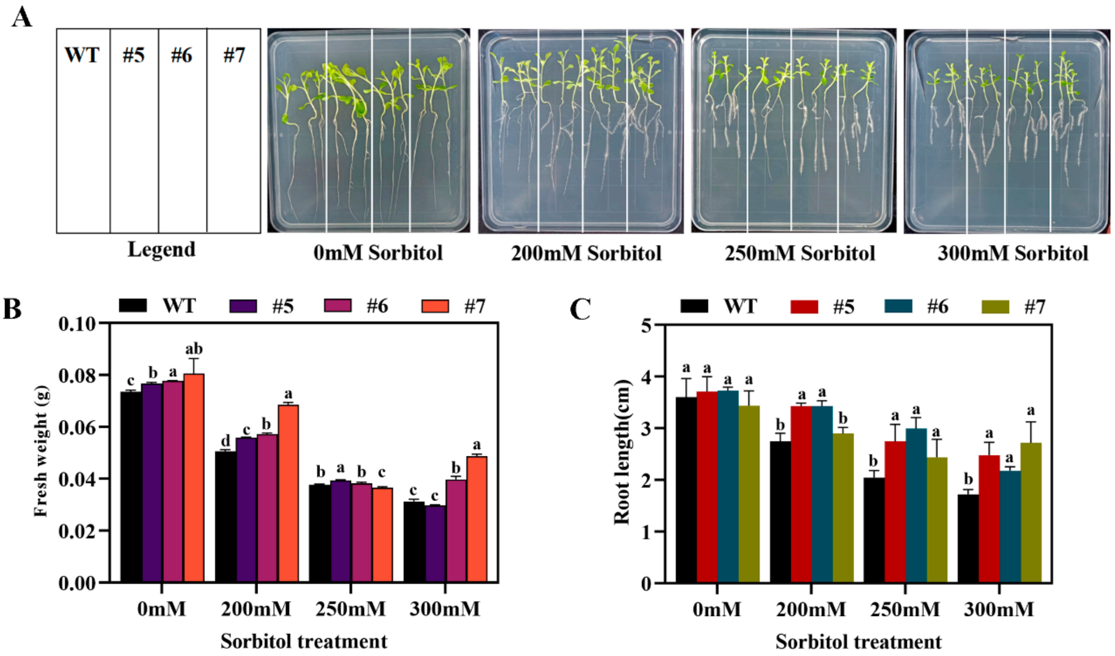

Supplementary Fig.S6 Phenotypic and physiological responses of transgenic *PuWRKY22* tobacco at the Three-Leaf Stage under Sorbitol stress. A: Phenotype of transgenic *PuWRKY22* tobacco lines ; B: Determination of fresh weight of transgenic *PuWRKY22* tobacco lines ; C: Determination of root length of transgenic *PuWRKY22* tobacco lines ; Note: Data are presented as mean ± SD; Bars labeled with different letters differ significantly (one-way ANOVA, Tukey’s HSD test, $p < 0.05$).

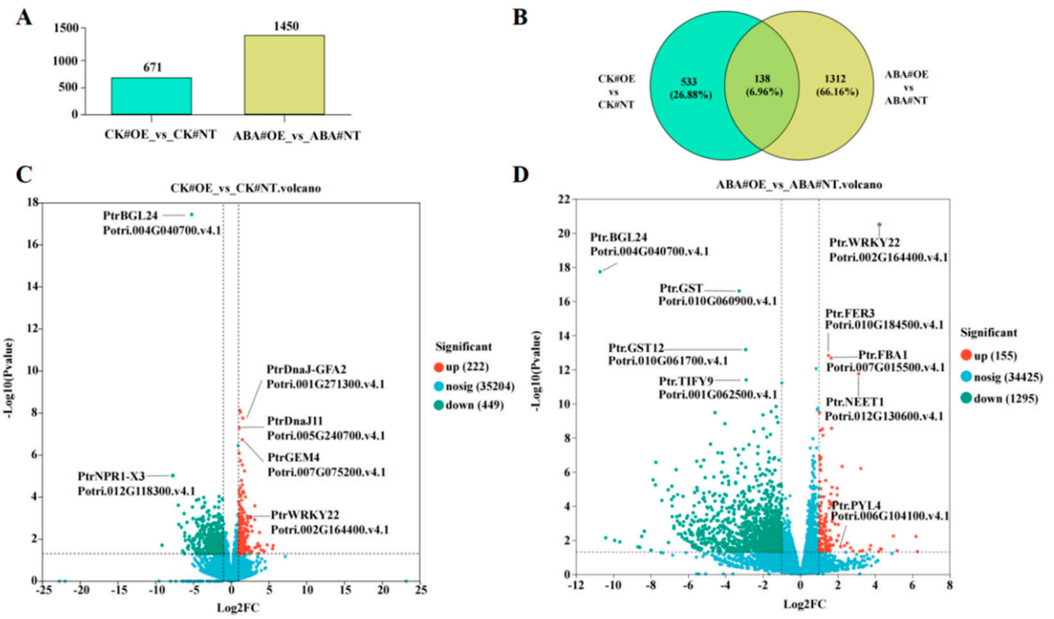

Supplementary Fig. S7. Identification of PuWRKY22-specific regulatory targets under ABA stress. A: Number of differentially expressed genes (DEGs) in comparisons CK#OE_vs_CK#NT and ABA#OE_vs_ABA#NT; B: Venn diagram of DEGs betweenCK#OE_vs_CK#NT and ABA#OE_vs_ABA#NT; C: Volcano plot of DEGs in CK#OE_vs_CK#NT; D: Volcano plot of DEGs in ABA#OE_vs_ABA#NT.
